# Metabolite Profiling and Transcriptome Analysis Unveil the Mechanisms of Red-Heart Chinese Fir [*Cunninghamia lanceolata* (Lamb.) Hook] Heartwood Coloration

**DOI:** 10.3389/fpls.2022.854716

**Published:** 2022-04-01

**Authors:** Sen Cao, Houyin Deng, Ye Zhao, Zijie Zhang, Yanting Tian, Yuhan Sun, Yun Li, Huiquan Zheng

**Affiliations:** ^1^National Engineering Laboratory for Tree Breeding, Key Laboratory of Genetics and Breeding in Forest Trees and Ornamental Plants of Ministry of Education, The Tree and Ornamental Plant Breeding and Biotechnology Laboratory of National Forestry and Grassland Administration, College of Biological Sciences and Technology, Beijing Forestry University, Beijing, China; ^2^Guangdong Provincial Key Laboratory of Silviculture, Protection and Utilization, Guangdong Academy of Forestry, Guangzhou, China

**Keywords:** Chinese fir, heartwood, metabolomic, flavonoid, transcriptomic

## Abstract

Red-heart Chinese fir (*Cunninghamia lanceolata*) has the advantages of high density and attractive color, making it popular in the market. To date, most studies about stems of woody plants have only been reported at the cytological level because of few living cells. In this study, the xylem was successfully partitioned into three effective sampling areas: sapwood, transition zone, and heartwood. Secondary metabolites, cell survival, and differentially expressed genes in the three sampling areas were, respectively, investigated. First, we identified the phenylpropanoid and flavonoid pathways closely related to color. Based on the chemical structure of secondary metabolites in pathways, two notable directions had been found. Luteolin’s glycosylation products might be the key substances that regulated the color of heartwood in red-heart Chinese fir because of the 1,000-fold difference between red-heart and white-heart. We also found pinocembrin and pinobanksin in Chinese fir, which were rarely reported before. At the cytological level, we believed that the transition zone of red-heart Chinese fir was a critical region for color production because of the fewer living ray parenchyma cells. In addition, transcriptome and quantitative reverse transcription PCR (qRT-PCR) proved that genes regulating the entire phenylpropanoid pathway, upstream of the flavonoid pathway, and some glycosyltransferases were significantly upregulated in the transition zone of red-heart and then colored the heartwood by increasing metabolites. This is the first report on the color-related secondary metabolites regulated by differential genes in red-heart Chinese fir. This study will broaden our knowledge on the effects of metabolites on coloring woody plant xylems.

## Introduction

The xylem of woody plants is not uniform and is divided into sapwood (SW) with living cells (5–25%), heartwood (HW) with dead cells, and transition zone (TZ) based on differences in color and cells (Taylor et al., 2002). HW has been a key area of metabolite research, due to it being concentrated with color-related secondary metabolites. However, due to the presence of mostly dead cells in HW, the TZ adjacent to it becomes the critical area to study for genes associated with metabolite production. In some species, the main feature of the TZ is the production of secondary metabolites which are produced in living ray parenchyma cells. In addition, with the loss of nuclear integrity and programmed cell death, metabolites accumulate and HW will be formed (Spicer, 2005). Therefore, studies on the regulatory mechanisms of secondary metabolites in HW should focus on the more active TZ.

Cells in the xylem usually lose their organelles and die after maturation (Funada et al., 2001), but the ray (radial), axial, and other specialized parenchyma cells will survive for several years or more in SW and TZ (Funada, 2000). One of the main characteristics of heartwood formation is the death of ray parenchyma cells. The ray parenchyma cells of Taiwania (*Taiwania cryptomerioides* Hayata) across the stem were found to die within 1–2 annual rings of the TZ (Chen et al., 2018). In addition, similar patterns were also found in other cytological studies of conifers. In *Abies sachalinensis*, the ray parenchyma cells undergo morphological changes, in which the organelles and, eventually, the nucleus disappear in the 8th to 10th annual ring from the cambium, respectively (Nakaba et al., 2006). Furthermore, in addition to the disappearance of the nucleus, heartwood formation in *Pinus densiflora* was accompanied by the disappearance of microtubules and vacuoles and nuclear DNA fragmentation (Nakaba et al., 2011).

It is widely accepted that flavonoids, which are polyphenolic compounds, include chalcones, flavanones, flavones, flavonols, and anthocyanins. They are synthesized from phenylalanine *via* the phenylpropanoid (*map00940*) and flavonoid (*map00941*) biosynthesis pathways which are known as the most thoroughly studied pathways (Jeong et al., 2004). The downstream of flavonoid metabolites are combined with glycans to produce glycosylated products. Given the value of secondary metabolites in HW, some studies have specifically focused on the color of HW, such as those in Norway spruce (*Picea abies*), Scots pine (*Pinus sylvestris*), and larch, etc. It not only makes wood products more attractive as the wood color also indirectly affects the decay resistance, durability, and mechanical strength of the wood, making it more available. Gierlinger et al. (2004a) concluded that the content of phenolic in larch heartwood was significantly correlated with the red color (*r* = 0.84). Similar patterns were reported for Douglas fir (Dellus et al., 1997a), *Juglans nigra* (Burtin et al., 1998), and European oaks (Mosedale et al., 1996). Moreover, a strong correlation was also found between the content of secondary metabolites (especially phenolics) and natural durability (Windeisen et al., 2002; Gierlinger et al., 2004b). Therefore, strong correlations among secondary metabolites content, heartwood color, and natural durability were anticipated. The relationship between color and natural durability is indirect and is based on the effects of the content of secondary metabolites on both factors. The measurement of wood color is advantageous in tree breeding and optimal utilization of heartwood, while the heritability of the production of metabolites could be high, implying a possibility of the change of heartwood color by the improvement of secondary metabolites through genetic breeding activity in woody plants (Fries et al., 2000; Ericsson et al., 2001).

Widely targeted metabolomic is a novel approach with high throughput, high sensitivity, and wide coverage that combines the advantages of non-targeted and targeted metabolomics. Especially, it had been successfully used to identify flavonoids and other secondary metabolites in crop, flower, and fruit species (Wu and Prior, 2005; Lin et al., 2011; Sun et al., 2012; Wang et al., 2019). Furthermore, the use of ultra-performance liquid chromatography-electrospray ionization tandem mass spectrometry (UPLC-ESI-MS/MS, hereafter, UEMS) in widely targeted metabolomic allows for the quantification of specific metabolites and the visual representation of metabolite differences.

Flavonoids are widely found in plants. Hence, the studies of their synthesis have also become a hot topic. Flavonoids and their derivatives are catalyzed by enzymes in pathways of phenylpropanoid and flavonoid. Firstly, phenylalanine produces naringenin that are sequentially catalyzed by phenylalanine ammonia-lyase (PAL), cinnamic acid 4-hydroxylase (C4H), and 4-coumarate CoA ligase (4CL). Then niringnin, an initiation substrate of the flavonoid pathway, produces flavonoid and its derivatives by using chalcone synthase (CHS), chalcone isomerase (CHI), flavanone 3-hydroxylase (F3H), flavonol synthase (FLS), flavanoe 3′-hydroxylase (F3′H) flavonoid 3′5′-hydroxylase (F3′5′H), flavone synthase I (FSI), and UDP-glycosyltransferase (UGT). While in some higher plant species, the MYB-bHLH-WD40 (MBW) transcription factor (TF) complex also regulates the flavonoid biosynthesis pathway. It contains an myeloblastosis (MYB) TF, a basic helix-loop-helix (bHLH), and a WD-repeat protein (Shirley, 2001; Zoratti et al., 2014; Xu et al., 2015). Among them, the R2R3-MYB TF plays an important role. Several R2R3-MYB family members have been shown to control flavonoid biosynthesis through various branches (Gierlinger et al., 2004a; Dubos et al., 2010; Jaakola, 2013).

Chinese fir [*Cunninghamia lanceolata* (Lamb.) Hook] is a fast-growing economic species in southern China which is also well known as a high-quality and -quantity wood with high compressive strength. The plantation area and stock volume of Chinese fir account for 1/4 and 1/3 of the total volume, respectively, both of which are the highest in China. Breeders had been working hard to cultivate high-quality and high-quantity Chinese fir varieties since 1976 (Zheng et al., 2013). Among them, the red-heart Chinese fir with a red color heartwood has the advantages of high density and attractive color, which is popular in the market (Duan et al., 2015). In the past, the research on Chinese fir with red heartwood was mainly focused on the collection and conservation of superior germplasms and the study of total extractives (Kui-Peng et al., 2017; Wen et al., 2018) with few studies on the classification of differential metabolites in different parts of xylem. Fan et al. (2015) found that content compounds in HW extracted by cold water, hot water, benzyl alcohol, ash, and 1% NaOH were higher than SW in red-heart Chinese fir. Two chemical compounds, namely, cedrol and sclareol, were extracted by ethanol and were more concentrated in HW than SW of red-heart Chinese fir (Yang et al., 2016). In addition, we have specifically classified the extracts through widely targeted metabolomic and identified that flavonoids were the main different metabolites in the HW of red-heart Chinese fir in an earlier study (Cao et al., 2020).

In this study, we explored the regulatory mechanism of flavonoids biosynthesis in colored heartwood at the metabolomic and transcriptomic levels. We focused on the differential expression of flavonoid metabolites and their regulatory genes in SW, TZ, and HW of red-heart and white-heart Chinese fir. While interpreting the functions of the TZ during heartwood formation, we not only demonstrated the critical role of TZ at the cytological level, but also highlighted the regulatory genes associated with flavonoid metabolites based on the correlation analysis between metabolomic and transcriptomic in TZ. Our results provided new ideas for the study of secondary metabolites in heartwood of woody plants. In addition, our results provided new insights into the occurrence of colored heartwood in red-heart Chinese fir and highlighted the usefulness of combined multi-omics analyses for understanding the biosynthetic mechanisms.

## Materials and Methods

### Plant Materials

**FIGURE 1:**
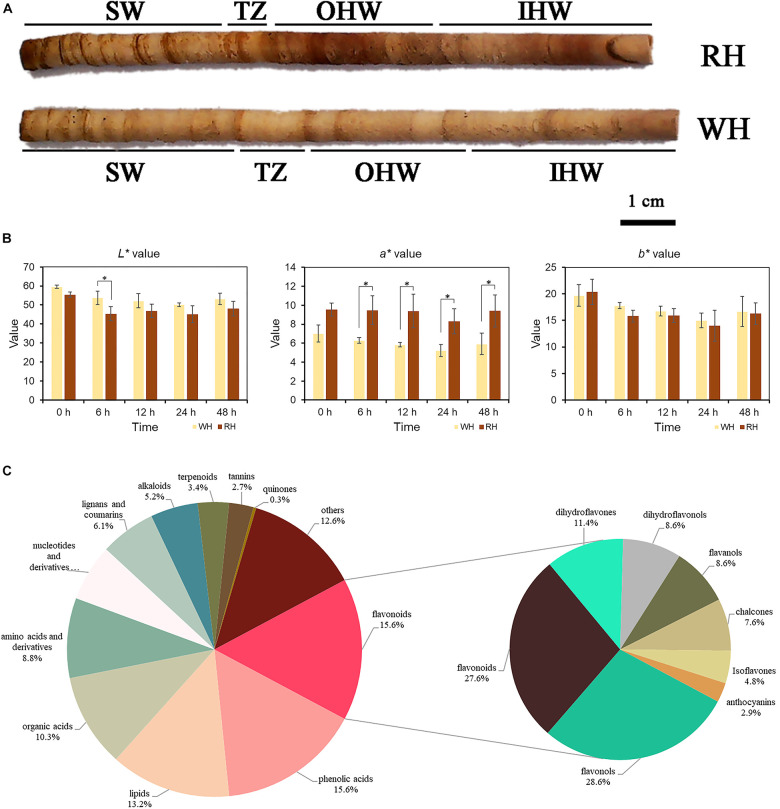
**(A)** A core of white-heart and red-heart Chinese fir wood, showing red-colored tissue (heartwood) and light-yellow tissue (sapwood), were divided into sapwood (SW), transition zone (TZ), outer heartwood (OHW), and inner heartwood (IHW) in the present study. The left side was closer to the pith. **(B)** L*a*b* values of white-heart and red-heart Chinese fir core after 0, 6, 12, 24, and 48 h *in vitro*. Data represent mean values ± SE of three independent measurements. *Indicates a significant difference between RH and WH at *p*-values < 0.05 by Duncan’s test. **(C)** Types and proportions of differential secondary metabolites among SW, TZ, OHW, and IHW in white-heart and red-heart Chinese fir.

Ten-year-old white-heart and red-heart Chinese fir samples belonging to clones cx110 and cx746, respectively, were collected as test material from Xiaokeng State Forest Farm (Guangdong, China, 24°70′N, 113°81′E). We sampled at 11:00–12:00 am on August 31, 2021 with a temperature of 29°C and humidity of 73–75%. In this study, wood core samples at breast height (1.3 m) were mainly selected as test material in each individual, and three individuals were randomly selected as biological replicates in clone cx110 and cx746, respectively. A total of three types of wood cores were selected for metabolomic, cytological, and transcriptomic studies, respectively. The wood samples were divided into SW, TZ, Outer heartwood (OHW), and Inner heartwood (IHW) (Figure 1A) for metabolomics; SW, TZ, and HW for cytological study; and Outer sapwood (OSW), Inner sapwood (ISW), and TZ for transcriptomics. The samples were stored at ambient temperature and humidity for metabolomics and immediately frozen in liquid nitrogen after obtaining materials, transported on dry ice, and eventually stored at −80°C for further use in transcriptomics.

### Color Measurement

The color of heartwood was measured at suitable humidity and temperature using a portable colorimeter (3nh NR10QC, Beijing, China). The colorimeter is an optical measuring instrument that simulates the human eye’s induction of red, green, and blue light. It is mainly based on the L*a*b* principle of the International Commission on Illumination (CIE) color space, measuring and displaying the color difference ΔE and ΔL*a*b* value between different samples. The CIE L*a*b* color system was used where L* describes the lightness, a* describes the chromatic coordinates on the green–red axes, and b* describes the blue–yellow axes, respectively. To determine the change in color of heartwood core *in vitro*, three measurements were taken from each heartwood core sample at 0, 6, 12, 24, and 48 h after separation from the stem. For each L, a and b values were measured three times and averaged.

### Metabolite Extraction, Identification, and Quantification

The specific experimental protocol of metabolite extraction was referred to the previous study (Cao et al., 2020). The samples were analyzed by the UEMS system after extraction. Based on the MWDB database of Wuhan (China) Mettewell Biotechnology Co., Ltd. and public databases, including MassBank,^1^ KNAPSAcK,^2^ HMDB,^3^ MoToDB,^4^ and METLIN,^5^ secondary metabolites detected by the UEMS system were compared and analyzed. Differential secondary metabolites were screened based on fold change ≥ 2 or ≤ 0.5 and thresholds of variable importance in projection (VIP) ≥ 1. On the other hand, principal component analysis (PCA) is the use of several principal components in a dimensionality reduction manner to explain the characteristics of the larger original data (Eriksson et al., 2013). A PCA diagram shows both the PC score of samples and the variable loadings. The PC represents the variables that explain most of the variation and are arranged in descending order.

### Light Microscopy

For the preparation of 10 um thick transverse sections, wood core samples were immediately immersed after collection in FAA (5 ml 38% formaldehyde, 5 ml glacial acetic acid, 90 ml 70% alcohol, and 5 ml glycerine). After returning to the lab, the wood cores were cut into sapwood, transition zone, and heartwood. After being embedded, the transverse sections were cut by a tungsten steel blade of the slicer (Leica HistoCore AUTOCUT) to the radial direction and stained with SafraninO-fast green for observation under light microscopes.

### Transcriptome Sequencing, Function Annotation, and Differentially Expressed Genes

Total RNA was extracted using a HiPure HP Plant RNA Mini Kit (Magen, R4165) and RNA integrity was determined by 1% agarose gel electrophoresis and Agilent Bioanalyzer 2100 (Agilent Technologies, Santa Clara, CA, United States). The library preparations were sequenced on an Illumina HiSeq 2500 platform and checked by Agilent 4200. The raw data were first filtered to remove contaminated adaptors and low-quality sequences. Then, the high-quality data were obtained by reassembling the sequences before being compared with public data. All assembled unigenes were aligned against the non-redundant National Center for Biotechnology Information (NCBI) protein database (Nr)^6^ (Zhang et al., 2018), Swiss-Prot,^7^ Gene Ontology (GO),^8^ clusters of orthologous groups (COGs),^9^ and Kyoto Encyclopedia of Genes and Genomes (KEGG).^10^

The fragments per kilobases per million reads (FPKM) values were read and analyzed for differential gene expression by evaluating their levels. In addition, log2 (fold change) ≥ 2 or ≤ 0.5 calculated using multiplicity of FPKM values were used as the threshold for screening differential genes. Meanwhile, the *p*-value was calculated by using false discovery rate (FDR) and *p*-value < 0.05 was set to be the threshold for screening significant level after multiple testing. Based on the KEGG pathways and gene functional annotation, DEGs regulating secondary metabolites synthases were finally identified.

### Real-Time Quantitative Reverse Transcription PCR

Total RNA was extracted using a HiPure HP Plant RNA Mini Kit (Magen, R4165). Reverse transcription was performed using PrimeScript™ RT Master Mix (TaKaRa). The primers used were designed using Primer Premier 5.0 software. The RT-qPCR was performed with an ABI 7500 Fast Real-Time Detection System. The amplification system consisted of 10.0 μl TB Green Premix Ex Taq II,0.4 μl of 10 μM forward primer,0.4 μl of 10 μM reverse primer,0.4 μl of ROX Reference Dye II (50 ×), 2 μl cDNA template, and 6.8 μl sterile distilled water for a total volume of 20 μl. The amplification program was 95°C for 30 s, followed by 40 cycles of 95°C for 5 s and 60°C for 34 s, 95°C for 15 s, and 60°C for 1 min. Relative quantitative analysis of data was performed with reference gene *EF1α* (Zhang et al., 2019). Primers for the quantitative reverse transcription PCR (qRT-PCR) are shown in Supplementary Table 1.

### Data Analyses

In this study, there were three biological replicates of each material. Microsoft Excel 2016 and IBM SPSS (version 24.0) were used for the data statistics, specifically for metabolites, FPKM value, etc. Differences in CIE L*a*b* values, metabolome, and transcriptome were assessed using analysis of variance (ANOVA), and multiple comparisons were performed using Duncan’s multiple range tests. ANOVA *t*-tests were considered statistically significant with *p*-values < 0.05.

## Results

### Color Measurement

The CIE L*a*b* values of heartwood in white-heart Chinese fir (WH) and red-heart Chinese fir (RH) after separation from the stem at 0, 6, 12, 24, and 48 h were measured. A significant relationship always existed in the a* value between WH and RH at 6, 12, 24, and 48 h wood core samples *in vitro* (Figure 1B). However, the a* value at 0 h had no significant difference. In addition, a significant difference only existed in L* value between WH and RH at 6 h compared to another period. Furthermore, there was no significant relationship in b* value between WH and RH in all periods. Color measurement at different periods after sampling found that no significant differences were observed in the L* and b* values, but there were significant relationships in the a* value between WH and RH at most periods. In addition, there were also differences in L*a*b* in the same sample at different periods. As the exposure time increased, the brightness seemed to show a significant downward trend in RH compared to the WH sample. The same trend was also found in the b* value. Despite this, there were no significant differences in a* value in RH at different periods, indicating that the exposure of the RH heartwood to the air did not make it more reddish.

### Metabolic Profiling of Sapwood, Transition Zone, Outer Heartwood, and Inner Heartwood Based on UPLC-ESI-MS/MS

To explain the color differences in various parts of the xylem, widely targeted metabolomic profiling was performed by using the UEMS system to investigate differences of secondary metabolites among SW, TZ, Outer heartwood (OHW), and Inner heartwood (IHW) in white-heart and red-heart Chinese fir. In general, a total of 673 secondary metabolites were identified in the present assay (SW, TZ, OHW, and IHW of white-heart and red-heart Chinese fir, Supplementary Table 2), including 105 flavonoids, 105 phenolic acids, 89 lipids, 69 organic acids, 59 amino acids and derivatives, 42 nucleotides and derivatives, 41 lignans and coumarins, 35 alkaloids, 23 terpenoids, 18 tannins, 2 quinones, and 85 others (Figure 1C). The most abundant metabolites in the present assay were flavonoids and phenolic acids, both of which accounted for 15.6% of the total secondary metabolites.

### Principal Component Analysis and Hierarchical Cluster Analysis

**FIGURE 2:**
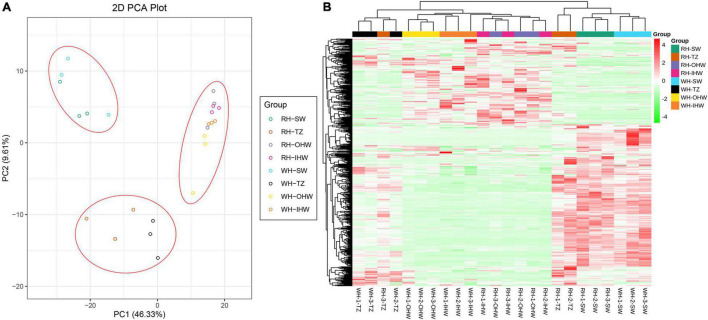
**(A)** PCA score plot of the metabolites among SW, TZ, OHW, and IHW of red-heart and white-heart Chinese fir. **(B)** Heatmap of secondary metabolites among SW, TZ, OHW, and IHW in white-heart and red-heart Chinese fir.

The metabolites were clearly separated into three groups according to the PCA analysis (Figure 2A). The PCA analysis of 24 samples revealed that the first principal component (PC1) and the second principal component (PC2) (with eigenvalues > 1) accounted for 55.94% of the total variance. In the PC1 × PC2 value plot, each sample was mainly separated in the PC1, which reached 46.33%. In addition, the results showed that three groups, namely, group SW, group TZ, and group OHW and IHW, were clearly grouped in PC1, and therefore distinguished the experimental sample locations. Furthermore, PC2 clearly distinguished SW, OHW, IHW, and TZ, which preliminary showed that there was a certain difference in secondary metabolites between the two groups. Furthermore, hierarchical cluster analysis (HCA) was introduced to further assess the differences in secondary metabolites among SW, TZ, OHW, and IHW. During which, it was found that metabolites were divided into three clusters by accumulating in different groups, thereby showing the different patterns of differential metabolite enrichment among different tissues (Figure 2B). Generally, the plot showed that secondary metabolites were significantly different and distinguishable in SW, TZ, and HW, but there was no evidence for differences in OHW and IHW. The results objectively illustrated the accuracy of this metabolomic data.

### Putative Metabolic Pathways for Differential Metabolites

**FIGURE 3:**
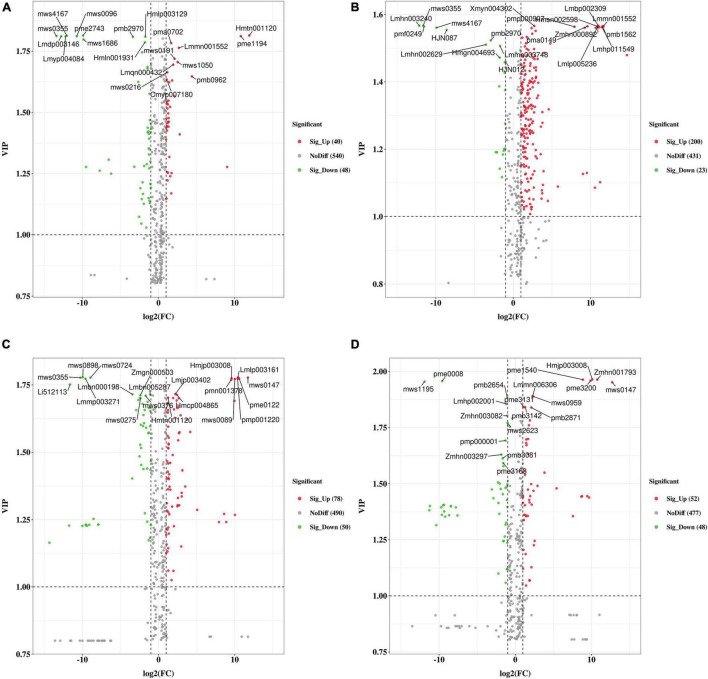
The volcano plot of the differential metabolites in SW, TZ, OHW, and IHW in white-heart and red-heart Chinese fir. **(A)** Differences in group WH-SW vs. RH-SW. **(B)** Differences in group WH-TZ vs. RH-TZ. **(C)** Differences in group WH-OHW vs. RH-OHW. **(D)** Differences in group WH-IHW vs. RH-IHW. Green dots represent downregulated metabolites, red spots represent upregulated metabolites, and gray dots represent insignificant differences in metabolites. The top 10 VIP values were marked, respectively.

We clustered the obtained differential metabolites according to the Kyoto Encyclopedia of Genes and Genomes (KEGG) database (see text footnote 10). These differential metabolites are mainly involved in the pathway-named biosynthesis of secondary metabolites (Supplementary Figure 1). In general, the metabolite comparisons among different groups showed different enrichment in KEGG. Particularly, flavonoid, flavone, and flavonol, which played essential roles in the flavonoid pathway, were significantly enriched in group WH-TZ vs. RH-TZ and WH-OHW vs. RH-OHW. Moreover, the criteria for identifying differential metabolites were VIP value ≥ 1 and fold-change scores of ≥ 2 or ≤ 0.5 among the metabolites. The screening results were analyzed using volcano plots (Figure 3). A total of 88, 223, 128, and 100 differential metabolites were identified in WH-SW vs. RH-SW, WH-TZ vs. RH-TZ, WH-OHW vs. RH-OHW, and WH-IHW vs. RH-IHW, respectively. Metabolites were more upregulated in red-heart Chinese fir than in white-heart Chinese fir of most groups, except in group WH-SW vs. RH-SW. The top 10 VIP values were separately marked in every volcano plot, indicating that only few metabolites were repeated in different groups (Supplementary Table 3). Most repeated metabolites were present in adjacent tissues, including Lmmn001552 in SW vs. TZ, mws0147 in OHW vs. IHW, and Hmjp003008 in OHW vs. IHW, which were upregulated in red-heart Chinese fir, and mws4167 in SW vs. TZ. Meanwhile, pmb2970 in SW vs. TZ was downregulated in red-heart Chinese fir. Particularly, Hmtn001120 was repeated in SW vs. TZ, while mws0355 was found in SW vs. TZ vs. OHW. On the one hand, this result showed that there was a significant specificity between tissues because of few repeated metabolites. On the other hand, differential metabolites also indicated that different metabolic reactions occurred in different wood tissues distributed in the radial direction.

### Widely Targeted Secondary Metabolite Assay for Flavonoids

The most abundant metabolites among these secondary metabolites were flavonoids. Based on a foldchange ≥ 2 or ≤ 0.5 and VIP ≥ 1, the number of differential flavonoids in WH-SW vs. RH-SW, WH-TZ vs. RH-TZ, WH-OHW vs. RH-OHW, and WH-IHW vs. RH-IHW were 21, 52, 24, and 19, respectively (Supplementary Table 4). These differentially expressed metabolites (DEMs) were summarized into 73 species, which were mainly classified into anthocyanins (3), Isoflavones (3), chalcones (6), dihydroflavones (7), dihydroflavonols (7), flavanols (9), flavonols (18), and flavonoids (20) (Supplementary Table 4). The number of upregulated flavonoids in each group was 9, 47,16, and 13, respectively. Interestingly, the ratio of upregulated flavonoids in WH-SW vs. RH-SW was 42.9%, significantly lower than 90.4% in WH-TZ vs. RH-TZ, 66.7% in WH-OHW vs. RH-OHW, and 68.4% in WH-IHW vs. RH-IHW, which means that the metabolites that affected the color of heartwood were more likely to be related to RH-TZ, RH-OHW, and RH-IHW. Taken together, by comparing the same parts in white-heart and red-heart Chinese fir, it was found that differences of flavonoids in TZ, OHW, and IHW seemed to be the key factor affecting heartwood color. In the following research, metabolites in WH-TZ vs. RH-TZ, WH-OHW vs. RH-OHW, and WH-IHW vs. RH-IHW will be conducted in a more in-depth analysis.

Comparing the SW, TZ, and HW of white-heart and of red-heart Chinese fir, respectively, it was found that flavonoid secondary metabolite profiles showed significant differences. Setting fold change ≥ 10 or ≤ 0.1 together with VIP ≥ 1 as thresholds for special differences, the contents of special differences between red-heart Chinese fir and white-heart Chinese fir are shown in Table 1, including 3 flavonoids, 3 flavonols, 3 chalcones, 4 dihydroflavones, and 2 dihydroflavonols.

**TABLE 1:** Differentially accumulated flavonoid compounds in the wood of “White-Heart” and “Red-Heart” Chinese fir.

Component name	Metabolite name	Content		Fold changes	VIP
		
		White-Heart	Red-Heart		
**FLAVONOIDS**					
WH-TZ vs. RH-TZ	Galangin	9.00E + 00	4.66E + 03	5.17E + 02	1.13
WH-OHW vs. RH-OHW	Luteolin-7-O-glucuronide-5-O-rhamnoside	9.00E + 00	6.94E + 03	7.71E + 02	1.77
	Luteolin-7-O-glucoside	9.00E + 00	3.53E + 03	3.92E + 02	1.27
WH-IHW vs. RH-IHW	Luteolin-7-O-glucuronide-5-O-rhamnoside	9.00E + 00	1.00E + 04	1.11E + 03	1.96
**FLAVONOLS**					
WH-TZ vs. RH-TZ	Kaempferol-3-O-robinoside-7-O-rhamnoside	9.00E + 00	5.41E + 03	6.01E + 02	1.56
WH-OHW vs. RH-OHW	Kaempferol-7-O-glucoside	9.00E + 00	9.37E + 03	1.04E + 03	1.77
WH-IHW vs. RH-IHW	Isorhamnetin-3-O-neohesperidoside	9.00E + 00	4.25E + 03	4.73E + 02	1.96
**CHALCONES**					
WH-TZ vs. RH-TZ	Naringenin chalcone	5.32E + 05	7.59E + 06	1.43E + 01	1.09
	Phloretin	1.12E + 04	1.53E + 05	1.36E + 01	1.31
	Isosalipurposide (Phlorizin Chalcone)	2.21E + 03	2.59E + 04	1.17E + 01	1.19
**DIHYDROFLAVONOES**					
WH-TZ vs. RH-TZ	Pinocembrin	9.00E + 00	1.41E + 06	1.56E + 05	1.05
	Butin	5.97E + 05	7.81E + 06	1.31E + 01	1.08
	Naringenin	8.49E + 05	1.07E + 07	1.26E + 01	1.05
	Naringenin-7-O-glucoside (Prunin)	3.05E + 03	3.66E + 04	1.20E + 01	1.27
**DIHYDROFLAVONOLS**					
WH-TZ vs. RH-TZ	Dihydrokaempferol-3-O-glucoside	9.00E + 00	1.72E + 04	1.92E + 03	1.56
	Pinobanksin	8.19E + 05	1.11E + 07	1.35E + 01	1.07

*WH-TZ, “Transition zone of White-heart Chinese fir”; RH-TZ, “Transition zone of Red-heart Chinese fir”; WH-OHW, “Outer Heartwood of White-heart Chinese fir”; RH-OHW, “Outer Heartwood of Red-heart Chinese fir”; WH-IHW, “Inner Heartwood of White-heart Chinese fir”; RH-IHW, “Inner Heartwood of Red-heart Chinese fir.” Metabolite fold changes, value > 1.0 represents increase; value < 1.0 represents decrease. Differentially accumulated flavonoid compounds were identified by threshold VIP (variable importance in projection) ≥ 1.0, and fold change ≥ 10 (upregulation) or ≤ 0.1 (downregulation). In order to get the correct value of fold changes, samples with a content of 0 were uniformly displayed as 9.00E + 00 in the table.*

Among the flavonoid compounds, both flavonoids (Galangin, Luteolin-7-O-glucuronide-5-O-rhamnoside, and Luteolin-7-O-glucuronide-5-O-rhamnoside) and flavonols (Kaempferol-3-O-robinoside-7-O-rhamnoside, Kaempferol-7-O-glucoside, and Isorhamnetin-3-O-neohesperidoside) showed the same trend of changes which were not detected in each part of white-heart Chinese fir. In contrast, most of chalcones (Naringenin chalcone, Phloretin, and Phlorizin chalcone), dihydroflavonoes (Butin, Naringenin, and Prunin), and dihydroflavonols (Pinobanksin) which were detected in white-heart Chinese fir were less than that in red-heart Chinese fir, with the same trend of changes as flavonoids and flavonols. In addition, differential flavonoid metabolites were mainly concentrated in the transition zone, while the types of metabolite that mainly belonged to flavonoids and flavonol in OHW and IHW were significantly less than in TZ (Table 1). Particularly, the content of Luteolin-7-O-glucuronide-5-O-rhamnoside was 770.80-fold higher in the WH-OHW vs. RH-OHW and 1,112.12-fold higher in the WH-IHW vs. RH-IHW. It was only flavonoid metabolite that exists in both comparison groups (Table 1).

### Differential Accumulation of Flavonoids Between White-Heart and Red-Heart Chinese Fir

**FIGURE 4:**
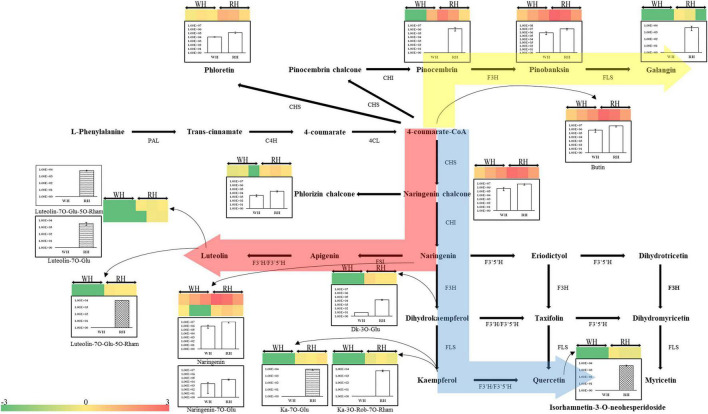
Biosynthetic pathway of flavonoids. This pathway is constructed based on the Kyoto Encyclopedia of Genes and Genomes (KEGG) pathway and literary references. WH, white-heart Chinese fir; RH, red-heart Chinese fir; DK, dihydrokaempferol; Ka, kaempferol; Glu, glucoside; Rham, rhamnose; Rob, robinoside; PAL, phenylalanine ammonia-lyase; C4H, cinnamate 4-hydroxylase; 4CL, 4-coumarate CoA ligase; CHS, chalcone synthase; CHI, chalcone isomerase; F3H, flavanone 3-hydroxylase; FLS, Flavonol synthase; F3′H, flavanone 3′-hydroxylase; F3′5′H, flavanone 3′-5′-hydroxylase. Each colored cell represents the normalized intensity of each compound ion according to the color scale (three biological replicates × two cultivars, *n* = 6). Column plots are shown for changes of flavonoids and their glycosylations in white-heart and red-heart Chinese fir. The empty column represents the WH-TZ vs. RH-TZ; the wave column represents the WH-OHW vs. RH-OHW; and the reticulate column represents the WH-ISW vs. RH-ISW.

The identified flavonoids and their glycosylations were arranged to their corresponding positions in the phenylpropanoid and flavonoid biosynthetic pathways. Figure 4 showed that the composition of compounds on the flavonoid biosynthesis pathways were specifically different depending on the Chinese fir cultivars (i.e., red-heart Chinese fir and white-heartwood Chinese fir) and tissues (i.e., TZ, OHW, and IHW). There were three main directions to synthesize downstream compounds. The synthesis of Galangin from Pinocembrin and Pinobanksin mainly shows significant differences in TZ between white-heart and red-heart. Specifically, Pinobanksin, which was different from Pinocembrin and Galangin, could also be detected in white-heart Chinese fir (Figure 4). In contrast, most of the different metabolites in the other two directions were glycosylated metabolites. The significant differences of luteolin glycosylation products mainly existed in OHW and IHW rather than TZ. In addition, luteolin glycosylations were not detected in white-heart Chinese fir. This means that Luteolin-7-O-glucuronide-5-O-rhamnoside and Luteolin-7-O-glucoside might have affected the color of heartwood in red-heart Chinese fir (Figure 4). The third direction was the most common flavonoid pathway from 4-coumarate-CoA to naringenin chalcone, naringenin, dihydrokaempferol, kaempferol, quercetin, and its glycosylations. The overall trend shows that the content of flavonoids and its glycosylations in red-heart Chinese fir was generally higher than in white-heart Chinese fir (Figure 4).

**FIGURE 5:**
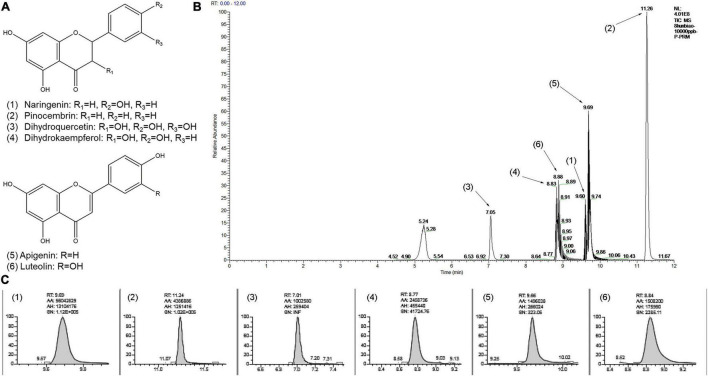
Quantification of several flavonoid metabolites by using high-performance liquid chromatography (HPLC). **(A)** Chemical structure of the flavonoid metabolites: (1) Naringenin, (2) Pinocembrin, (3) Dihydroquercetin, (4) Dihydrokaempferol, (5) Apigenin, (6) Luteolin. **(B)** The chromatogram of the mixed standards by HPLC. **(C)** The chromatogram of the flavonoid metabolites of Chinese fir samples by using HPLC.

Furthermore, the quantification of several flavonoid metabolites in heartwood of red-heart and white-heart Chinese fir were executed, and a total of six metabolites were determined (Figure 5 and Supplementary Table 5). In general, the amount of naringenin, dihydroquercetin, and dihydrokaempferol showed the difference between red-heart and white-heart Chinese fir. Among them, the content of naringenin was significantly higher than the other five metabolites. These results intuitively elucidated the difference in the content of flavonoid metabolites between red-heart and white-heart Chinese fir.

### Transcriptome Analysis

**FIGURE 6:**
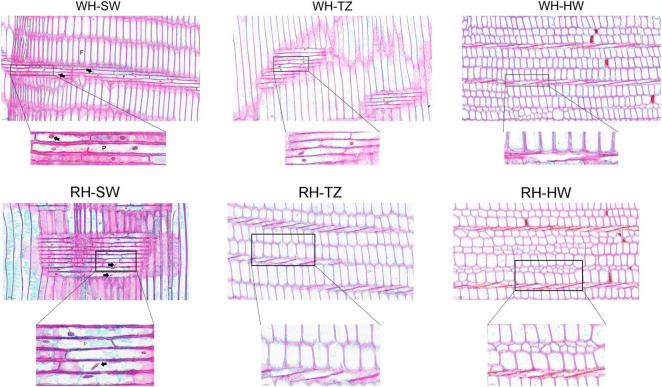
Radial sections of ray parenchyma cells in the SW, TZ, and HW of white-heart and red-heart Chinese fir. F, fiber; P, ray parenchyma cells. The complete nucleus was indicated by the arrow.

By slicing the three radially distributed areas (SW, TZ, and HW) and staining the nucleus with SafraninO-fast green, it was found that the heartwood containing a large amount of secondary metabolites were dead cells, and only the ray parenchyma cells in the sapwood were living cells, in which complete nuclei could be observed (Figure 6). However, the ray parenchyma cells in WH-TZ were all living cells, but the cells in RH-TZ were almost all dead, indicating that the ray parenchyma cells in RH-TZ might die earlier than in WH-TZ. Furthermore, the shape of the nuclei in the RH-SW ray parenchyma cells were elliptical rather than the standard round shape like the nuclei in WH-SW (Figure 6). Despite this, there seemed to be no literature explaining why this appeared.

Through observation, it was found that the heartwood consisted dead cells. Therefore, we cannot perform RNA-seq. Hence, sapwood and transition zone were used in the study of the transcriptome. RNA-Seq produced 38,275,319, 37,317,278, 36,242,635, 38,484,952, 35,759,742, and 37,330,818 clean reads from RH-TZ, RH-ISW, RH-OSW, WH-TZ, WH-ISW, and WH-OSW libraries, respectively. Three hundred thousand eight hundred two final unigenes were identified from clean data of the 18 libraries (3 replicates for each sample) with 490.08 base pairs (bp) in average length. In particular, the N25 value was 1,319 bp and the N50 value was 519 bp.

**FIGURE 7:**
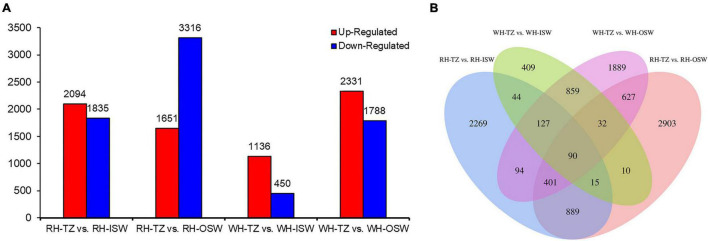
Statistic of differentially expressed genes. **(A)** The number of upregulated and downregulated differentially expressed genes (DEGs) in group RH-TZ vs. RH-ISW, RH-TZ vs. RH-outer sapwood (OSW), WH-TZ vs. WH-inner sapwood (ISW), and WH-TZ vs. WH-OSW. **(B)** Venn diagram analysis of DEGs. RH, red-heartwood; WH, white-heartwood; TZ, transition zone; ISW, Inner Sapwood; OSW, Outer Sapwood. Differentially expressed genes were identified by *p* ≤ 0.05 and the absolute value of log2 ratio ≥ 2.

There were 3,929, 4,967, 1,586, and 4,119 DEGs in the four comparison groups (RH-TZ vs. RH-ISW, RH-TZ vs. RH-OSW, WH-TZ vs. WH-ISW and WH-TZ vs. WH-OSW, respectively, Figure 7A and Supplementary Table 6). Compared TZ, ISW, and OSW in red-heart and white-heart Chinese fir, 2,094 and 1,651 unigenes were upregulated, while 1,835 and 3,316 unigenes were downregulated in RH-TZ vs. RH-ISW, RH-TZ vs. RH-OSW, respectively. In addition, 1,136 and 2,331 unigenes were upregulated in WH-TZ vs. WH-ISW and WH-TZ vs. WH-OSW, both exceeding the number of downregulated unigenes in this two comparison groups. Venn diagram analysis revealed the presence of 90 DEGs in all four comparison groups (Figure 7B). In addition, 889 DEGs were commonly found in both RH-TZ vs. RH-ISW and RH-TZ vs. RH-OSW, and 2,269 DEGs were only found in RH-TZ vs. RH-ISW. GO analysis assigned 46,763, 20,446, and 47,908 unigenes to the biological process, cell component, and molecular functional class, respectively. The metabolic process in the biological process contained the largest number of unigenes (Supplementary Figure 2). The clusters of orthologous groups of protein database (COG) annotation allocated 129,056 unigenes into 25 COG categories (Supplementary Figure 3). The general function prediction only identified 17,094 unigenes (13.25%) and RNA processing and modification (16,456 unigenes, 12.75%) were the largest groups, followed by signal transduction mechanism (12,971 unigenes, 10.05%). KEGG analysis revealed metabolism, genetic information processing, environmental information processing, cellular processes, and organismal systems as the changed pathways in groups. Specifically, carbohydrate metabolism contained the most quantity (Supplementary Figure 4).

### Transcription Factors

**FIGURE 8:**
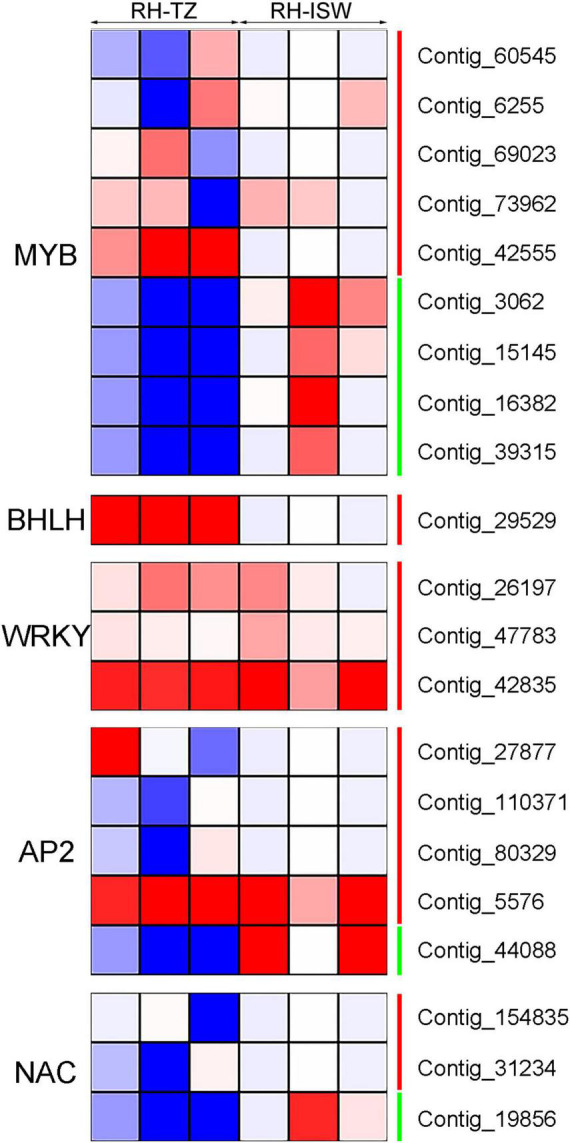
Differentially expressed transcription factors in RH-TZ vs. RH-ISW. The green line on the right side of the heatmap represented downregulation while the red line represented upregulation. Differentially expressed transcription factors were identified by *p* < 0.05 and absolute value of log2 ratio ≥ 2.

**FIGURE 9:**
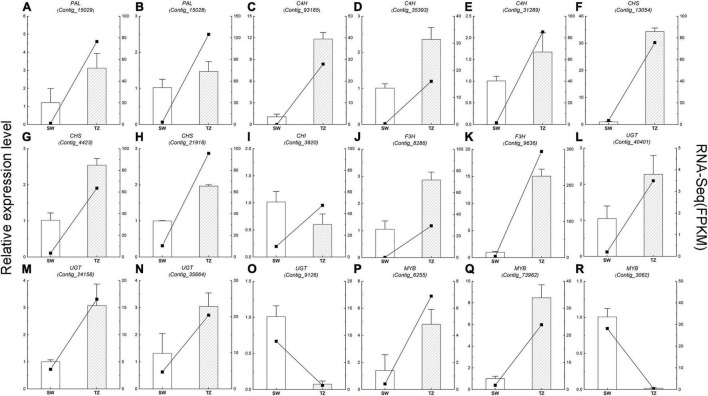
The qRT-PCR analysis of the expression level and fragment per kilobase per million reads (FPKM) of 18 phenylpropanoid and flavonoid biosynthetic pathways candidate unigenes in RH-SW and RH-TZ. The column showed the relative expression level while the line showed the FPKM value. **(A,B)** Phenylalanine ammonia-lyase (PAL), **(C–E)** cinnamic acid 4-hydroxylase (C4H), **(F–H)** chalcone synthase (CHS), **(I)** chalcone isomerase CHI, **(J,K)** flavanone 3-hydroxylase (F3H), **(L–O)** UDP-glycosyltransferase (UGT), and **(P–R)** MYB.

There were 21 differentially expressed transcription factors identified in RH-TZ vs. RH-ISW. The differentially expressed transcription factors were annotated to encode MYB, bHLH, WRKY, AP2/ERF, and NAC (Figure 8). Nine *MYB* factors were differentially expressed, while 5 genes were found upregulated. The other 4 were downregulated in RH-TZ. In addition, all of the bHLH and WRKY transcription factors were upregulated in RH-TZ. Only *Contig_44088* in AP2 and *Contig_19856* in NAC were found downregulated in the AP2 and NAC families. We further performed RT-PCR to verify the correctness of the differential transcription factors in RNA-Seq. MYB TFs (*Contig_6255*, *Contig_73962*, and *Contig_3062*) were found to have the same trend in RT-PCR and FPKM (Figure 9).

### Phenylpropanoid and Flavonoid Biosynthetic Pathways

**FIGURE 10:**
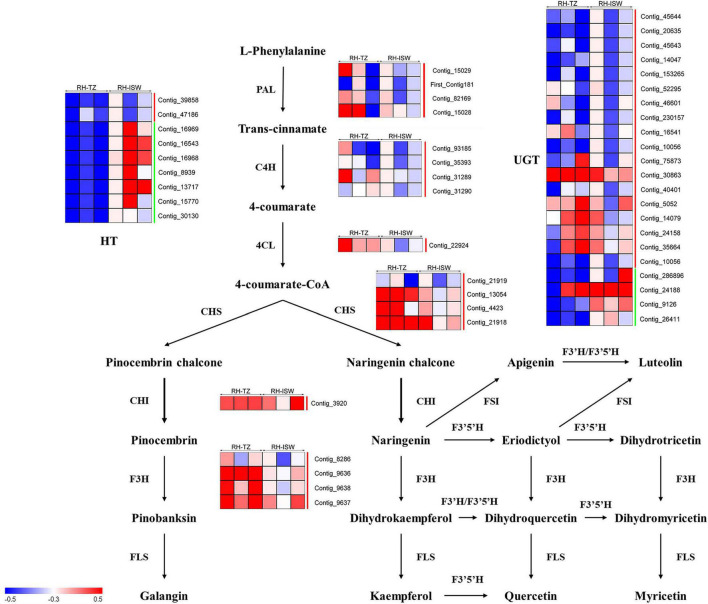
Transcript profiling of genes in the phenylpropanoid and flavonoids biosynthetic pathways in RH-TZ vs. RH-ISW of red-heart Chinese fir. RH, red-heart Chinese fir; TZ, transition zone; ISW, Inner Sapwood; PAL, phenylalanine ammonia-lyase; C4H, cinnamic acid 4-hydroxylase; 4CL, 4-coumarate CoA ligase; CHS, chalone synthase; CHI, chalcone isomerase; F3H, flavanone 3-hydroxylase; FLS, flavonol synthase; F3′H, flavanoe 3′-hydroxylase; F3′5′H, flavonoid 3′5′-hydroxylase; FSI, flavone synthase I. The green line on the right side of the heatmap represented downregulation while the red line represented upregulation.

In Figure 10, except for the DEGs hexosyltransferase (HT), most of the unigenes in phenylpropanoid and flavonoid biosynthetic pathways were upregulated. Only two HT unigenes (*Contig_39858* and *Contig_47186*) were upregulated, while the other seven genes were downregulated. In addition, another glycosyltransferase gene named UGT was also found in RH-TZ vs. RH-ISW, of which 18 of the gene were upregulated and 4 downregulated (*Contig_286896*, *Contig_24188*, *Contig_9126*, and *Contig_26411*). Considering that the main roles of UGT and HT unigenes were to bind glycosides to different sites of flavonoids, which enriched the types of flavonoid derivatives present, the results indicated that the downstream genes of the flavonoids pathway in the transition zone have higher expression. *PAL* (*Contig_15029*, *First_Contig181*, *Contig_82169*, and *Contig_15028*), *C4H* (*Contig_93185*, *Contig_35393*, *Contig_31289*, and *Contig_31290*) and *4CL* (*Contig_22924*) were upregulated in the phenylpropanoid pathway. Since the phenylpropanoid pathway was upstream of the flavonoid pathway, the upregulation of key genes in RH-TZ increased the amount of substrates for the flavonoid pathway, thereby promoting the production of flavonoid metabolites. Furthermore, the upstream genes in the flavonoid pathway, such as *CHS* (*Contig_21919*, *Contig_13054*, *Contig_4423*, and *Contig_21918*), *CHI* (*Contig_3920*), and *F3H* (*Contig_8286*, *Contig_9636*, *Contig_9637*, and *Contig_9638*), were also upregulated in RH-TZ vs. RH-ISW. In general, most of the differential genes in RH-TZ vs. RH-ISW were upregulated in the phenylpropanoid and flavonoid biosynthetic pathways. They mainly existed in the phenylpropanoid pathway, the upstream of the flavonoid pathway, and the downstream of flavonoid pathway which aimed to produce flavonoid glycosylation, but there was no significant difference in the expression of unigenes, such as FLS, FSI, and F3′5′H, in flavonoid pathway.

### Quantitative Reverse Transcription PCR Validation of the Transcriptomic Data

The expression of 18 DEGs (5 phenylpropanoid biosynthetic pathway unigenes, 10 flavonoid biosynthetic pathway unigenes, and 3 transcription factor unigenes) in RH-ISW and RH-TZ were detected, and their relative expression levels were analyzed in SW and TZ using qRT-PCR (Figure 9 and Supplementary Table 7). The expression patterns of these unigenes were similar to the FPKM of RNA-Seq results. Among them, expression patterns of 17 unigenes (14 unigenes upregulated and 3 unigenes downregulated) were similar to FPKM, while *CHS* (*Contig_3920*) showed different patterns. The results demonstrated a strong correlation of data between FPKM and RT-PCR, with both showing good agreement for both upregulated and downregulated gene expression. In Figure 9, the expression level of *CHS* (*Contig_13054*) in RH-TZ was 33 times higher than in RH-SW. Besides, the expression level of *PAL* (*Contig_93185*) and *F3H* (*Contig_9636*) was 12 and 15 times higher in TZ, respectively. This demonstrated that the major DEGs in RH-TZ were concentrated in the upstream of the phenylpropanoid and flavonoid biosynthesis pathways. From the qRT-PCR results, three MYB TFs (*Contig_6255*, *Contig_73962*, and *Contig_3062*) were found to be significantly different. Considering that MYB TFs play key regulatory roles in a variety of plant functions, the three TFs were clustered with other MYBs associated with flavonoid biosynthesis pathway in other species, including *Arabidopsis thaliana*, *Malus domestica*, *Salvia miltiorrhiza*, *Gerbera hybrida*, *Solanum lycopersicum*, *Daucus carota*, and strawberry, and a phylogenetic tree was drawn (Supplementary Figure 5). In the phylogenetic tree, only *Contig_6255* was found to be strongly related to the other MYBs which was associated with flavonoid synthesis, compared to the other two TFs (*Contig_73962* and *Contig_3062*).

## Discussion

In this study, secondary metabolites, cell survival, and DEGs in the SW, TZ, and HW of xylem were, respectively, investigated. We rapidly obtained the information of 673 metabolites in SW, TZ, OHW, and IHW through the extensive target metabolomics analysis method based on the UEMS system, and accurately classified them in Chinese fir. Metabolic pathway analyses and types of metabolites revealed that the flavonoid biosynthesis pathway was significantly different in WH-SW vs. RH-SW, WH-TZ vs. RH-TZ, WH-OHW vs. RH-OHW, and WH-IHW vs. RH-IHW. In addition to the deeper color, more flavonoids were also contained in red-heart Chinese fir rather than in white-heart Chinese fir. Among secondary metabolites, color substrates had been widely studied in horticulture and agriculture because of its aesthetic, nutritional, and medicinal properties. Metabolomic analysis revealed significant differences in *Cerasus humilis* fruit color, accompanied by differences in 11 metabolites (Ji et al., 2020). Wang et al. (2021) found that flavonoid metabolites showed great differences among the different colored safflower, specifically, C-glycosylquinochalcones. In addition, there were also studies about color substrates on other fruit trees (Baldi et al., 2018; Li et al., 2018; Zhang et al., 2020). In this study, we identified several differential flavonoids in TZ, OHW, and IHW of red-heart Chinese fir compared to white-heart. More importantly, a significant accumulation of flavonoids (Galangin, Luteolin-7-O-glucuronide-5-O-rhamnoside, and Luteolin-7-O-glucuronide-5-O-rhamnoside), flavonols (Kaempferol-3-O-robinoside-7-O-rhamnoside, Kaempferol-7-O-glucoside, and Isorhamnetin-3-O-neohesperidoside), chalcones (Naringenin chalcone, Phloretin, and Phlorizin chalcone), dihydroflavonoes (Butin, Naringenin, and Prunin), and dihydroflavonols (Pinobanksin) was revealed (Table 1 and Figure 4). Additionally, we identified the phenylpropanoid and flavonoid biosynthetic pathways closely related to color. Based on the chemical structure of secondary metabolites in pathways, they are divided into three directions, including two notables. On the one hand, luteolin and its glycosylation products were the secondary metabolites with the largest difference in content in this study. Its content in red-heart Chinese fir was 1,000 times that of white-heart. Therefore, we supposed that luteolin and its glycosylation products might be the key substances that regulated the color of the heartwood in red-heart Chinese fir. On the other hand, we found pinocembrin and pinobanksin in Chinese fir, which were rarely reported before. It was found that flavonoids showed significant differences between TZ and HW of red-heart and white-heart Chinese fir. Similar findings were also shown in the study of Douglas fir. Dihydroquercetin was identified and showed a significant difference between SW and HW by using HPLC and nuclear magnetic resonance (NMR) in Douglas fir. Its resulting unstable leucocyanidin was found in heartwood after spontaneously auto-oxidizing to polymeric pigments (Dellus et al., 1997a,b). It was found that taxifolin and dihydrokaempferol were significantly different by exploring the distribution of heartwood metabolites in hybrid parents (Pâques et al., 2013). In another study, 71 kinds of isoflavones and flavonoids were found in HW and SW and showed significant difference by metabolomic profiling in *Taxus chinensis* (Pilger) (Shao et al., 2019). Sugar conjugation to the flavonoid aglycone increases the solubility, stability, and bioavailability in contrast to flavonoid aglycones (Plaza et al., 2014). Flavonoids represent the major molecules involved in plant pigmentation. As a downstream product of the flavonoid pathway, anthocyanidins are highly unstable and easily susceptible to degradation. Therefore, glycosylation is essential to stabilize them and serve as a signal for transport of the anthocyanins to vacuoles where they can function as pigments (Zhang et al., 2020). It is reasonable to assume that the two luteolin glycosylation products which are chemically similar to the anthocyanins may directly or indirectly affect the color of red-heart Chinese fir. Unlike the flavonoid metabolites in heartwood of most conifers, the secondary metabolites in *Cryptomeria japonica* and *Sequoia sempervirens* heartwood were norlignans (NorL), a class of secondary metabolites with diphenylpentane C6-C5-C6 carbon skeletons (Zhang et al., 2005; Yanase et al., 2015). Sequirin-C and agatharesinol, two kinds of Norls, were significantly detected in the heartwood of *Cryptomeria japonica* and might be associated with the darkening of the heartwood (Takahashi and Mori, 2006; Nakaba et al., 2016).

In addition to the difference in color, the living cells also show significant differences in the radial distribution of the stem, which mainly manifested closer to the pith, the fewer living cells, with the rupture of the nucleus, and the reduction of energy substances, such as starch. At the cytological level, we suggest that the transition zone of red-heart Chinese fir was a critical region for color production because of the fewer living ray parenchyma cells in RH-TZ. In addition to the early observation of living ray parenchyma cells and the partial disappearance of nuclei and lipids ranging from sapwood to heartwood in *C. japonica*, more cytological studies of ray parenchyma cells were conducted in several conifers (*Pinus densiflora*, *Pinus rigida*, and *Abies sachalinensis*) by Nakaba et al. (2008). In them, a new hypothesis was proposed which could show the pattern of differentiation and cell death in the ray parenchyma cells and their adjacent ray tracheids. Starch disappeared earlier and cells died earlier in ray tracheids than ray parenchyma cells (Nakaba et al., 2008). The observation of ray parenchyma cells and ray tracheids indicated that the position effect determined their function in the formation of heartwood. In this study, nuclei were only found in the ray cells of SW and TZ in red-heart and white-heart Chinese fir and not in HW. Furthermore, cells were dead when near the pith and living cells were less in RH-TZ rather than in WH-TZ. This aimed to demonstrate that ray parenchyma cells in RH-TZ might be related to the color change of heartwood. In HW, secondary metabolites can be released from cellular and subcellular regions upon cell-death (Celedon and Bohlmann, 2018). The released phenolics which synthesized in parenchyma cells diffuse into adjacent cell walls and lumens and give heartwood its specific color (Dejardin et al., 2010). Studies in *Cryptomeria japonica*, using a combination of light microscopy and mass-spectrometry, suggested that the diterpenoid molecule ferruginol accumulates in axial and ray parenchyma from where it may diffuse into heartwood tracheids (Imai et al., 2005). Phenolic secondary metabolites in the form of glycosides can be stored in large quantities in vacuoles. The aglycons can be released by glycosidases, which may come in contact with glycosides when the integrity of cellular or subcellular regions breaks down during parenchyma cell-death (Celedon and Bohlmann, 2018). On the basis of the differential metabolites, we focused on the expression of unigenes in phenylpropanoid and flavonoid biosynthetic pathways of SW and TZ, which have also been demonstrated to upregulate the expression of unigenes that synthesize various flavonoids and their glycosylation in the transition zone. The phenylpropanoid and flavonoid biosynthetic pathways had been widely researched while the phenylpropanoid pathways are upstream of the flavonoid pathway. Tian et al. (2021) found that *PAL* and *C4H* were significantly different between the leaves of a novel golden variety in *Populus deltoides* and the normal *Populus deltoides*. They aimed to demonstrate how the regulatory genes involved in naringenin, a substrate for flavonoid synthesis, were upregulated in the golden variety. In addition, CHS and CHI were also the early enzymes involved in the biosynthetic pathway of flavonoids and had been shown to upregulate and participate in the synthesis of flavonoid metabolites in a variety of plants, such as potato (Wang et al., 2018), strawberry (Baldi et al., 2018), and Actinidia arguta (Li et al., 2018). In our study, *PAL*, *C4H*, *CHS*, *CHI*, and *F3H* in RH-TZ had been found upregulated rather than in RH-SW. This showed the accumulation of precursor substances of color components in the heartwood of red-heart Chinese fir (Figure 10). In addition, UGTs were well known to be a vital enzyme for anthocyanin synthesis and had been widely reported in flowers and fruits, including pomegranate (Qin et al., 2020), jujube (Zhang et al., 2020), and grape (Yang et al., 2020). In red-heart Chinese fir, the high expression of *UGT* unigenes indicated that flavonoid metabolites were being transduced into their glycosylation productions, which might be the key color substance that caused the heartwood to turn red (Figure 9). The MYB TF family had been demonstrated to act as the main flavonoid biosynthesis regulator in many plant species. In *Arabidopsis*, *MYB4* was suggested to play dual roles in modulating the flavonoid biosynthetic pathways (Wang et al., 2017). In Malus crabapple, *MdMYB8* was found to regulate flavonol biosynthesis, and that auxin, ethylene, abscisic acid (ABA), and jasmonic acid (JA) might also be involved in this process (Li et al., 2020). Furthermore, *DcMYB113* was found to activate the expression of its bHLH partner and the anthocyanin biosynthetic structural genes (Xu et al., 2020). In our study, nine MYB TFs were identified, and three of them (*Contig_6255*, *Contig_73962*, and *Contig_3062*) had the same pattern with FPKM in RH-TZ vs. RH-SW by qRT-PCR analysis. After phylogenetic analysis, only *Contig_6255* may be associated with flavonoid synthesis in differential TFs. In addition, its function needs to be further verified in future work. qRT-PCR results also showed that PAL (*Contig_93185*), CHS (*Contig_13054*), and F3H (*Contig_9636*) differed most significantly between RH-TZ and RH-SW, indicating that the differential expressions were mostly concentrated in the upstream of the phenylpropanoid and flavonoid biosynthetic pathways (Figure 10).

## Conclusion

Most of the extractives of Chinese fir had been widely reported for a long time, while single metabolites and synthesis mechanism had not been deeply studied. In this study, flavonoid metabolites were considered to be the main differential metabolites in white-heart and red-heart Chinese fir. Through the metabolome, it was found that there were significant differences in the content of metabolites in the radial distribution of stems in Chinese fir. In addition, by slicing the SW, TZ, and HW of the wood core, it was found that the ray parenchyma cells gradually died as they were closer to the pith, resulting to how the heartwood formation is to be accompanied by the death of ray parenchyma cells. Furthermore, transcriptome analysis of living ray parenchyma cells showed that the gene expression reached the peak in the transition zone, rather than SW, the upstream, and downstream differential genes. In addition, TFs were significantly found in phenylpropanoid and flavonoid biosynthetic pathways. The specific regulation of different members in a gene family leads to the binding of metabolites to specific chemical groups, resulting in the difference of metabolite types. We expected that our work on flavonoid metabolites and their genes will not only promote the study of secondary metabolites in Chinese fir wood, but also provide some basis for further study on the phenylpropanoid and flavonoid biosynthetic pathways. To further study the genes and TFs on flavonoids the synthesis and regulation of flavonoids metabolites, the further verification of the function of related upstream genes and glycosyltransferases may be important. Hence, it is worthy of additional in-depth study. This is the first report on secondary metabolites that determine the color of heartwood regulated by differential genes in red-heart Chinese fir. This study will broaden our knowledge on the effects of metabolites on the coloring of woody plant xylems and provide a theoretical basis for cultivating high-quality red-heart Chinese fir in the future.

## Data Availability Statement

The datasets presented in this study can be found in online repositories. The names of the repository/repositories and accession number(s) can be found below: We have uploaded the data on GSA data base at https://ngdc.cncb.ac.cn/, and the GSA accession number is CRA005929.

## Author Contributions

YL and HZ conceived and designed the experiments. SC wrote the article. SC, HD, YZ, ZZ, and YT performed the experiments and analyzed the data. YS participated and helped to complete the experiments. All authors contributed to the article and approved the submitted version.

## Conflict of Interest

The authors declare that the research was conducted in the absence of any commercial or financial relationships that could be construed as a potential conflict of interest.

## Publisher’s Note

All claims expressed in this article are solely those of the authors and do not necessarily represent those of their affiliated organizations, or those of the publisher, the editors and the reviewers. Any product that may be evaluated in this article, or claim that may be made by its manufacturer, is not guaranteed or endorsed by the publisher.

## Footnotes

1
http://www.massbank.jp/

2
http://kanaya.naist.jp/KNApSAcK/

3
http://www.hmdb.ca/

4
http://ted.bti.cornell.edu/cgi-bin/TFGD/metabolite/compound.cgi

5
http://metlin.scripps.edu/index.php

6
http://www.ncbi.nlm.nih.gov

7
http://www.expasy.ch/sprot

8
http://www.geneontology.org

9
http://www.ncbi.nlm.nih.gov/COG/

10
http://www.genome.ad.jp/kegg/

## Supplementary Material

Supplementary Figure 1 Overview of pathway analysis of sapwood (SW), transition zone (TZ), outer heartwood (OHW), and inner heartwood (IHW) in white-heart and red-heart Chinese fir. **(A)** Differences in pathway of WH-SW vs. RH-SW. **(B)** Differences in pathway of WH-TZ vs. RH-TZ. **(C)** Differences in pathway of WH-OHW vs. RH-OHW. **(D)** Differences in pathway of WH-IHW vs. RH-IHW.

Supplementary Figure 2 Gene Ontology (GO) function annotation diagram.

Supplementary Figure 3 Clusters of orthologous groups (COGs) diagram.

Supplementary Figure 4 Kyoto Encyclopedia of Genes and Genomes (KEGG) diagram.

Supplementary Figure 5 Phylogenetic relationship of *Contig_6255*, *Contig_73962*, *Contig_3062*, and other MYBs. The genes used for alignment and phylogenetic analysis were *Arabidopsis thaliana* (*AtMYB4* NM_120023, *AtMYB11* NM_116126, *AtMYB12* NM_130314, *AtMYB75* NM_104541, *AtMYB90* NM_105310, *AtMYB111* NM_124310, *AtMYB113* NM_105308, *AtMYB114* NM_105309, *AtMYB123* NM_122946), *Malus domestica* (*MdMYB1* GU270471, *MdMYB6* HM122631, *MdMYB8* DQ267899), *Salvia miltiorrhiza* (*SmMYB1* MT188153), *Gerbera hybrida* (*GhMYB1* NM_001326832), *Solanum lycopersicum* (*SlMYB75* NM_001279063), *Daucus carota* (*DcMYB113* MK896875), and strawberry (*FaMYB10* MN689832).
