# Supplementary figures and images for: Metabolite Profiling and Transcriptome Analysis Unveil the Mechanisms of Red-Heart Chinese Fir [Cunninghamia lanceolata (Lamb.) Hook] Heartwood Coloration
Source: Front Plant Sci. 2022 Apr 1;13:854716. doi: 10.3389/fpls.2022.854716 (PMC9022624; doi:10.3389/fpls.2022.854716)

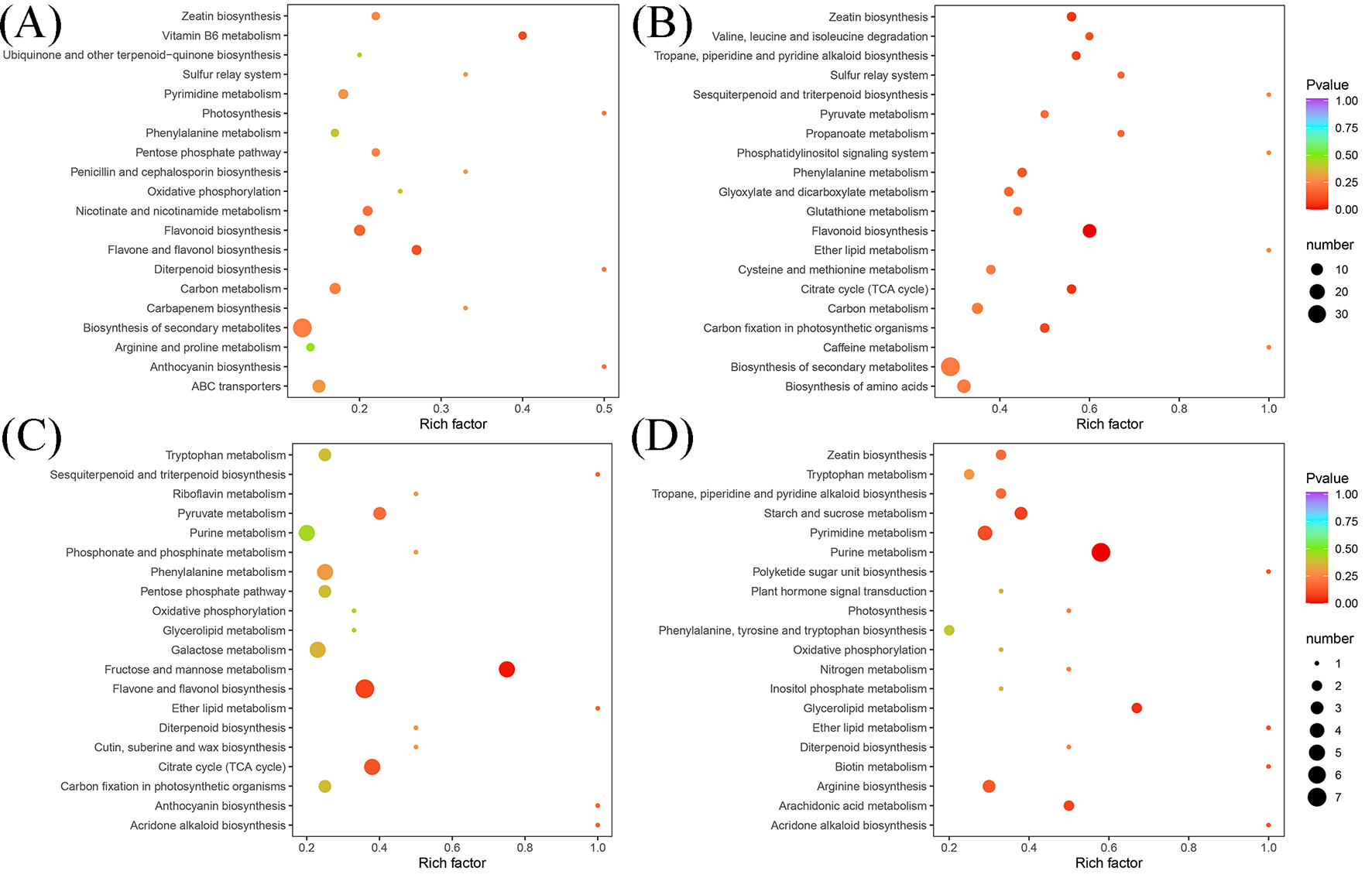

Supplement: Supplementary Figure 1 — Overview of pathway analysis of sapwood (SW), transition zone (TZ), outer heartwood (OHW), and inner heartwood (IHW) in white-heart and red-heart Chinese fir. (A) Differences in pathway of WH-SW vs. RH-SW. (B) Differences in pathway of WH-TZ vs. RH-TZ. (C) Differences in pathway of WH-OHW vs. RH-OHW. (D) Differences in pathway of WH-IHW vs. RH-IHW. [file Image_1.TIF]

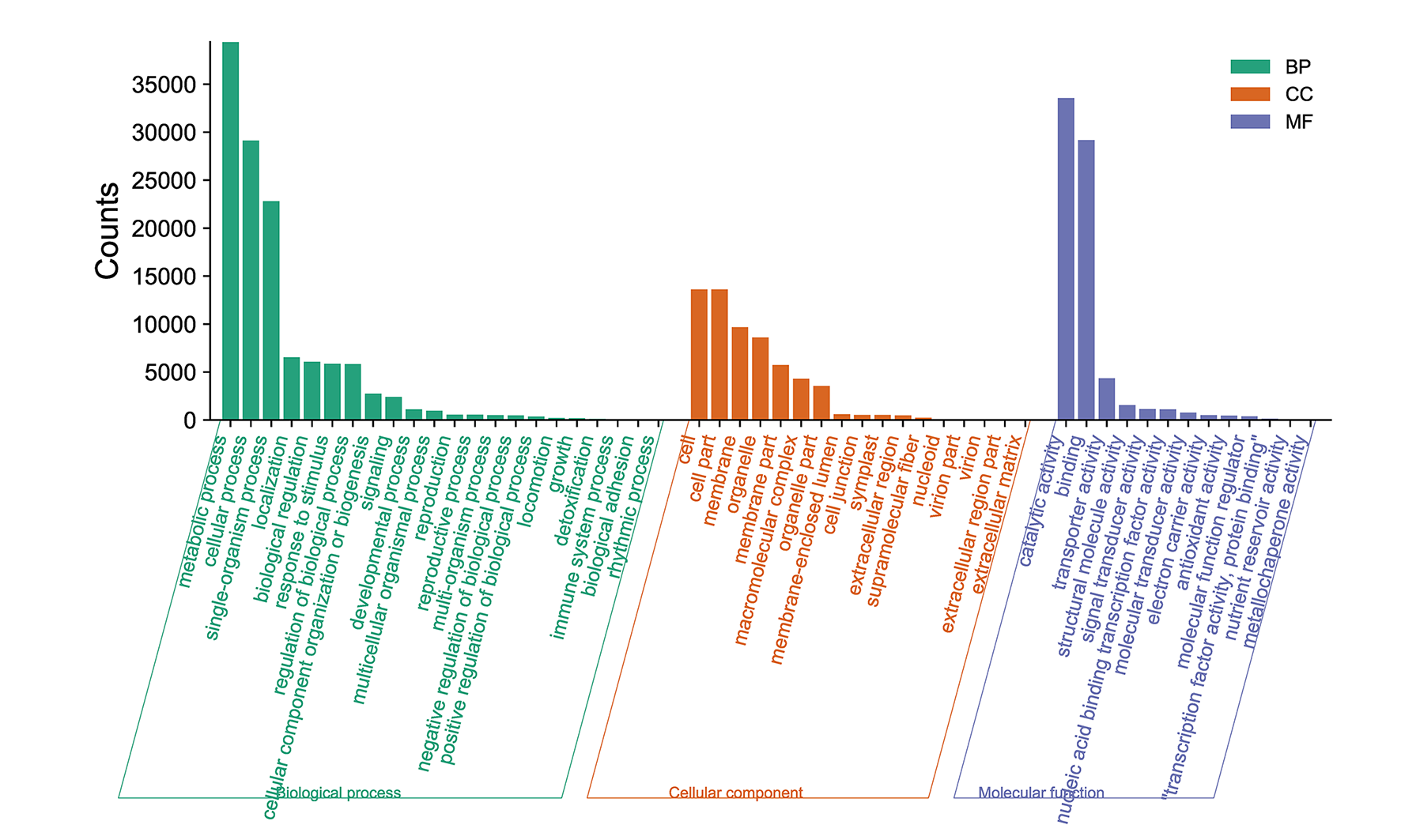

Supplement: Supplementary Figure 2 — Gene Ontology (GO) function annotation diagram. [file Image_2.TIF]

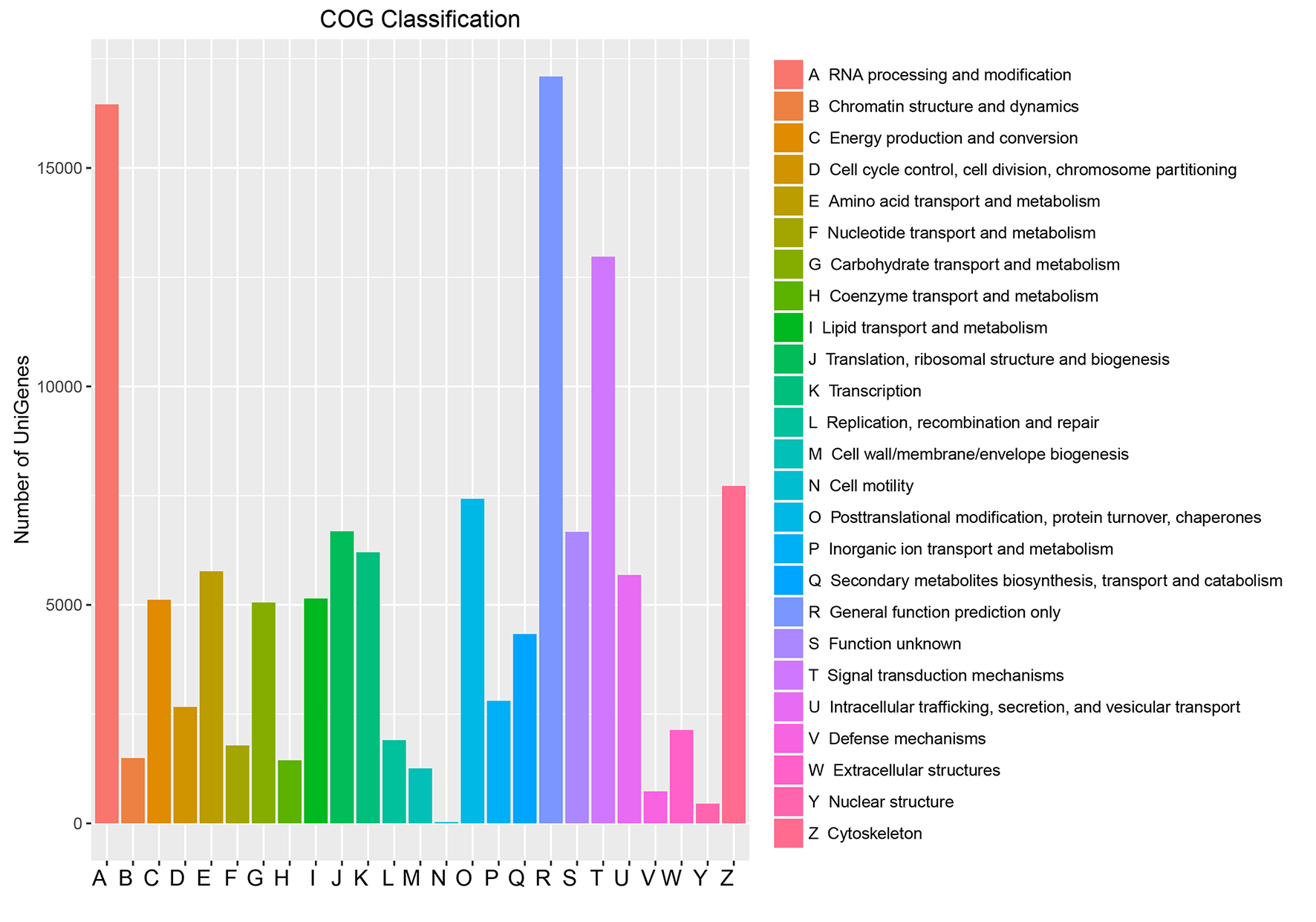

Supplement: Supplementary Figure 3 — Clusters of orthologous groups (COGs) diagram. [file Image_3.TIF]

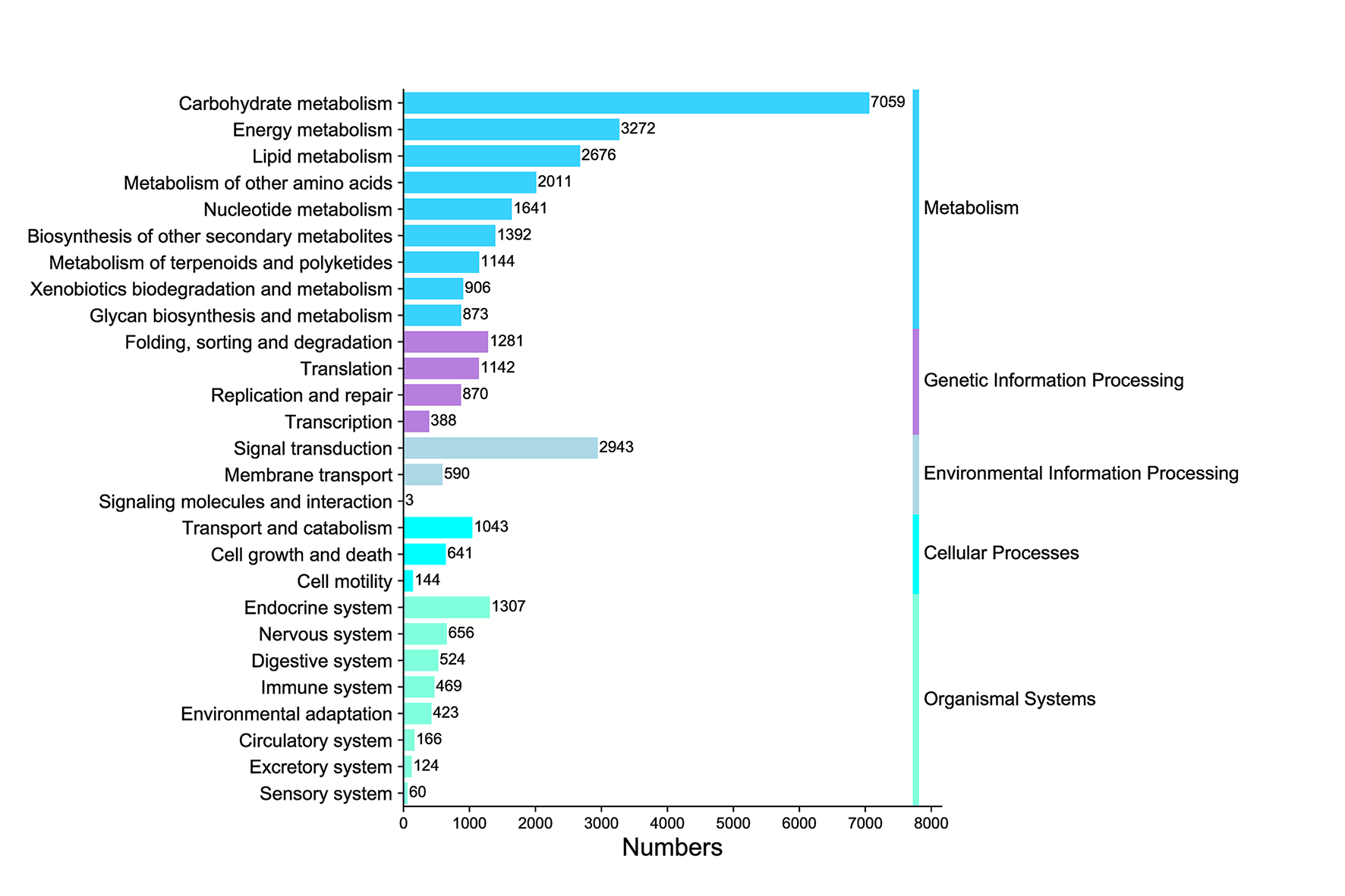

Supplement: Supplementary Figure 4 — Kyoto Encyclopedia of Genes and Genomes (KEGG) diagram. [file Image_4.TIF]

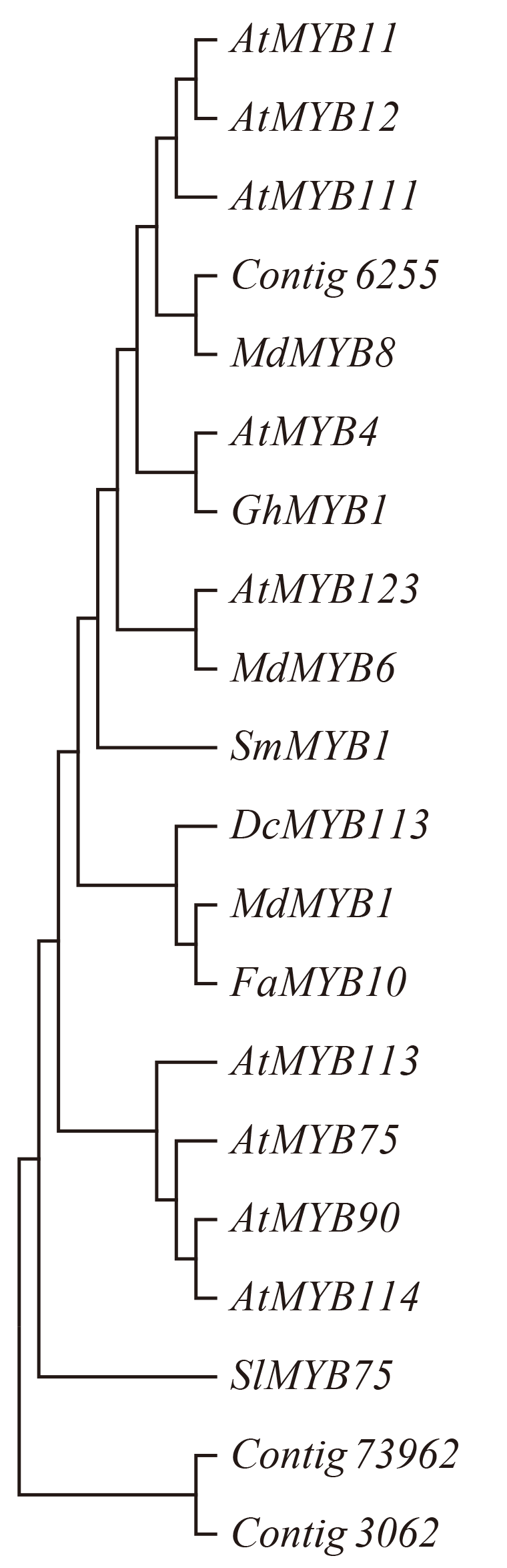

Supplement: Supplementary Figure 5 — Phylogenetic relationship of Contig_6255, Contig_73962, Contig_3062, and other MYBs. The genes used for alignment and phylogenetic analysis were Arabidopsis thaliana (AtMYB4 NM_120023, AtMYB11 NM_116126, AtMYB12 NM_130314, AtMYB75 NM_104541, AtMYB90 NM_105310, AtMYB111 NM_124310, AtMYB113 NM_105308, AtMYB114 NM_105309, AtMYB123 NM_122946), Malus domestica (MdMYB1 GU270471, MdMYB6 HM122631, MdMYB8 DQ267899), Salvia miltiorrhiza (SmMYB1 MT188153), Gerbera hybrida (GhMYB1 NM_001326832), Solanum lycopersicum (SlMYB75 NM_001279063), Daucus carota (DcMYB113 MK896875), and strawberry (FaMYB10 MN689832). [file Image_5.TIF]
